# Systemic amyloidosis manifestation in a patient with psoriatic arthritis^☆^^☆☆^

**DOI:** 10.1016/j.abd.2020.07.012

**Published:** 2021-03-15

**Authors:** Bruno de Castro e Souza, Camila Fátima Biancardi Gavioli, Walmar Roncalli Pereira de Oliveira, Ricardo Romiti

**Affiliations:** Dermatology Department, Universidade de São Paulo, São Paulo, SP, Brazil

**Keywords:** Psoriasis, Psoriatic arthritis, Systemic amyloidosis

## Abstract

Systemic amyloidosis secondary to psoriatic arthritis is rare, and published data are based mainly on case reports and are associated with increased mortality. This is the report of a patient with long-term psoriatic arthritis and chronic sialadenitis, who showed an inadequate response to therapy. The diagnosis of secondary amyloidosis was attained through biopsies of genital skin lesions. Although very rare, it is important that dermatologists and general practitioners consider the possibility of amyloidosis in patients with chronic inflammatory diseases, since an early intervention can be implemented, and thus, the prognosis of this condition can be improved.

## Introduction

Systemic amyloidosis is a disease characterized by the extracellular deposition of insoluble fibrils produced by a soluble precursor protein.^1^, ^2^, ^3^ These fibrils have an affinity for the Congo red dye, which shows a characteristic green birefringence when viewed under polarized light.^1^, ^2^, ^3^ Four types of systemic amyloidosis are most frequently seen: AL (caused by clonal plasma cell dyscrasia), AA (caused by inflammatory conditions), ATTR (caused by mutations of the precursor protein transthyretin), and Ab2M amyloidosis (caused by end-stage renal disease).^1^, ^2^, ^3^

AA amyloidosis is caused by long-term inflammation, such as arthritis, inflammatory bowel disease, and chronic infections.^3^ The precursor of this type of amyloidosis is the serum amyloid A (SAA) apolipoprotein, an acute phase reagent.^2^ Kidney disease, autonomic neuropathy, intestinal involvement, splenomegaly, hepatomegaly, goiter, and cardiomyopathy are the most common signs of this type of amyloidosis.^1^, ^2^, ^3^ Oral involvement has been previously reported, whereas skin lesions are not common.^4^

Type AA amyloidosis that complicates psoriatic arthritis is rare, and the published data are primarily based on case reports and are associated with increased mortality.^5^, ^6^ An early diagnosis of systemic amyloidosis is usually attained through an aspiration biopsy of abdominal subcutaneous fat. However, in cases of psoriatic arthritis, the diagnosis usually seems to be late and happens at the kidney disease stage, due to the rarity of amyloidosis in this rheumatological condition.^6^

This is a case report of a patient with long-term psoriatic arthritis and chronic sialadenitis, with an inadequate response to multiple therapies. The diagnosis of secondary amyloidosis was attained through biopsies of genital skin lesions. There were no signs of kidney failure.

## Case report

A 55-year-old white woman came to the Dermatology outpatient clinic complaining of darkened lesions in the axillae, vulva, and perineal region for 3 years. The patient had psoriatic arthritis and typical nail alterations caused by psoriasis (nail pitting and hyperkeratosis) since she was 11 years old, with significant motor sequelae. She had been treated with leflunomide and etanercept for 9 years, but her arthritis became progressively worse. She also had recurrent bilateral anterior uveitis for seven years and xerostomia due to chronic sialadenitis for 3 years.

**Figure 1:**
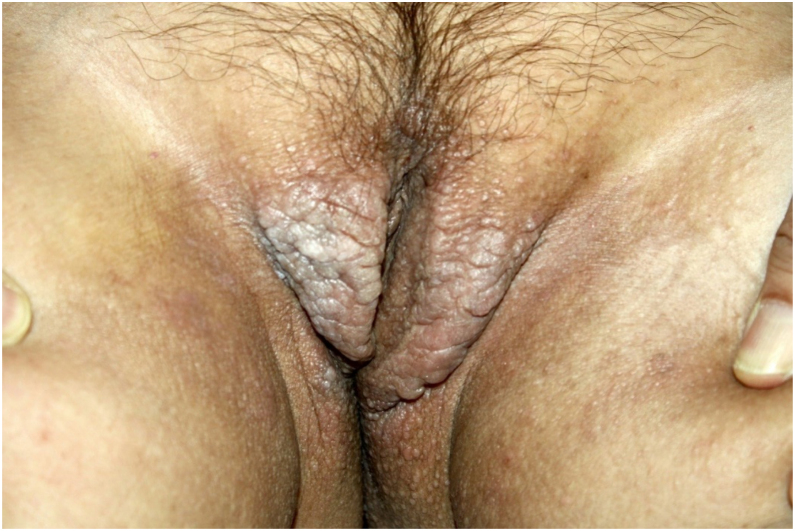
Infiltrated brown plaques on the vulva.

**Figure 2:**
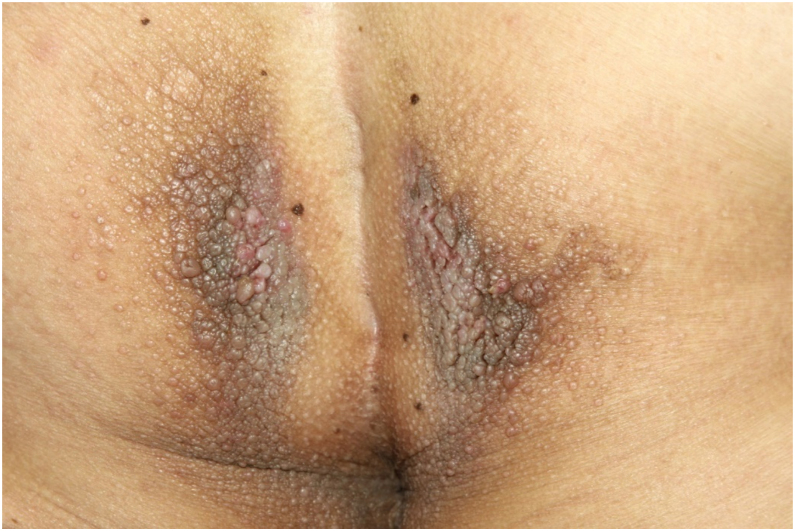
Confluent brown papules and plaques on the perianal area.

**Figure 3:**
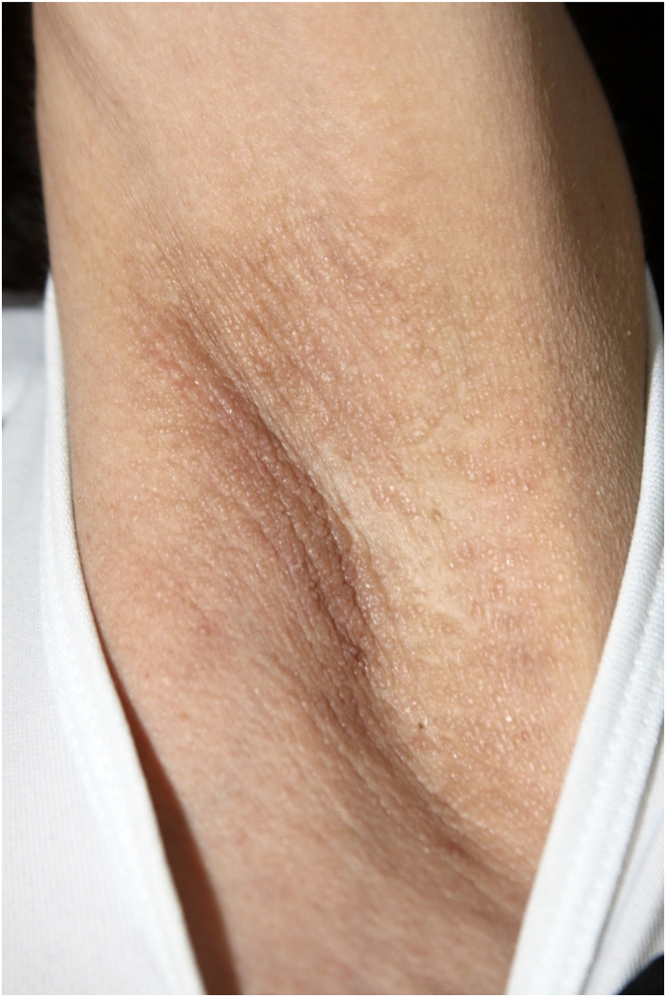
Brownish papules on the axilla.

On physical examination, papules converged into brown plaques infiltrated in the vulvar and perianal area and on the axillae (Figure 1, Figure 2, Figure 3). The joint examination showed symmetrical joint deformity in the hands.

Laboratory tests showed anemia, with hemoglobin of 10.7 g/dL; total serum proteins: 7.1 g/L; serum albumin: 3.1 g/L; serum globulin: 4.0 g/L; ESR: 96 mm/h. Protein electrophoresis showed an increase in gamma globulins. Immunoglobulins showed: IgG 1,808 mg/dL; IgA 6.0 mg/dL; IgM 84.3 mg/dL. The 24 -h urine collection showed normal results. The levels of creatinine, serum amylase, and lipase were normal. The bone densitometry showed widespread bone density loss.

**Figure 4:**
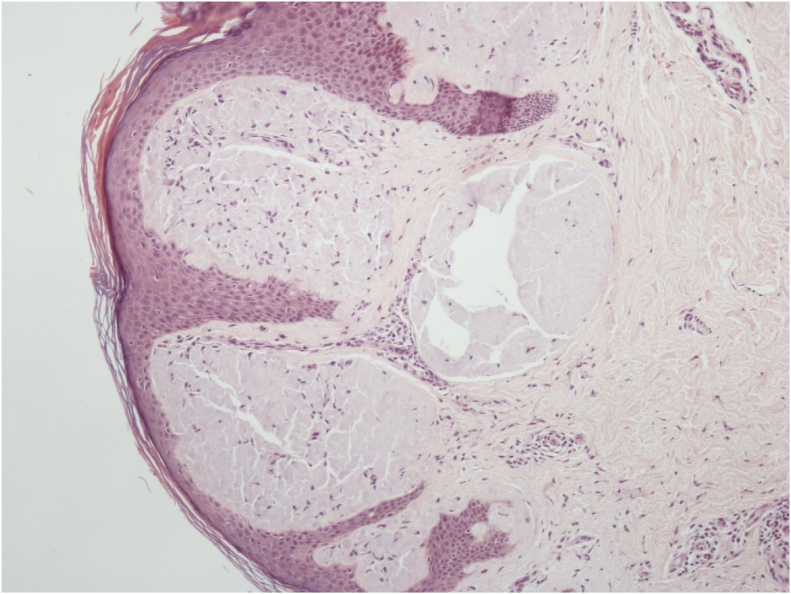
Deposit of amorphous hyaline substance in the dermis.

**Figure 5:**
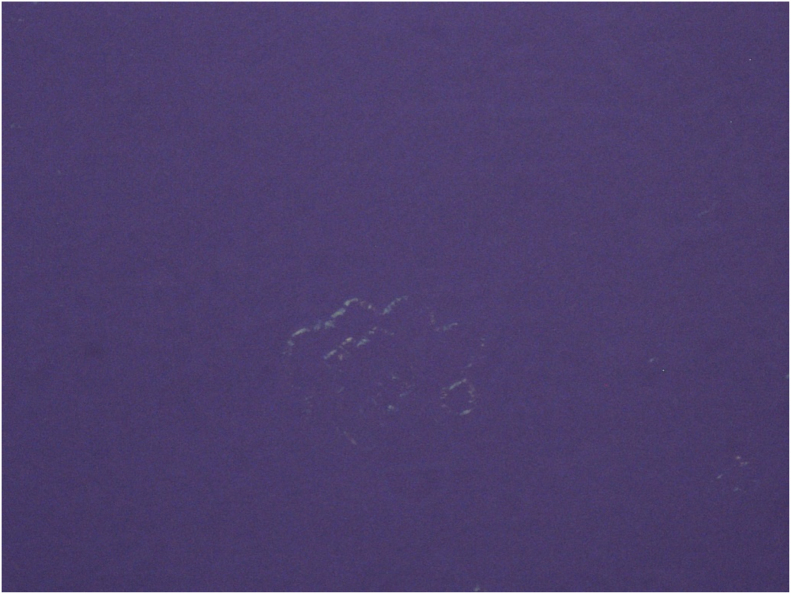
Green birefringence under polarized light in Congo red stained tissue.

Histopathological analysis of a vulvar papule and of salivary gland tissue showed deposits of amorphous hyaline substance that stained positively with Congo red and were birefringent when seen under polarized light, confirming the diagnosis of systemic amyloidosis at both sites (Figure 4, Figure 5).

## Discussion

Systemic amyloidosis has an incidence of approximately eight per million people per year.^4^ AA amyloidosis is more common than the other forms, which can be justified by its association with infectious diseases, especially tuberculosis.^3^ In the Western world, AA amyloidosis has become rarer due to bacterial infection control. However, other chronic inflammatory conditions have replaced infections as the most common cause.^3^

Skin lesions, usually seen in primary systemic amyloidosis, are rare in the secondary form of the disease. In the literature review, some cases of oral and skin involvement were found in AA amyloidosis.^4^, ^5^

The etiopathogenesis of amyloidosis is not clear. Inflammatory conditions lead to cytokine expression, particularly of interleukin six, which causes the liver to overproduce SAA protein.^3^ SAA protein is usually degraded by monocyte-derived enzymes, so in most cases, it does not lead to amyloidosis. Therefore, patients with secondary amyloidosis have an enzyme defect and also a genetically-determined structural abnormality in the SAA protein, causing resistance to degradation.^4^

The definitive diagnosis of systemic amyloidosis is made through biopsy and staining with Congo red, through demonstration of apple-green birefringence under polarization microscopy.^3^ Primary cutaneous amyloidosis does not show apple-green birefringence under polarization microscopy.

AA amyloidosis therapy aims to reduce the production of SAA. Currently, the available treatments are corticosteroids, cytostatic drugs, and monoclonal antibody cytokines (TNF and IL-6).^3^, ^6^, ^7^, ^8^, ^9^, ^10^ Our patient developed secondary systemic amyloidosis during treatment of the underlying disease with etanercept, which shows that the disease was extremely aggressive in this case. Therefore, the early diagnosis was essential to prevent damage to other organs.

Although very rare, it is crucial that dermatologists and general practitioners consider the possibility of systemic amyloidosis in patients with chronic inflammatory diseases, since an intervention can be implemented early and the prognosis of this condition can be improved.

## Financial support

None declared.

## Authors’ contributions

Bruno de Castro e Souza: Drafting and editing of the manuscript; critical literature review.

Camila Fátima Biancardi Gavioli: Data collection, analysis, and interpretation; drafting and editing of the manuscript; critical literature review.

Walmar Roncalli Pereira de Oliveira: Study conception and planning; drafting and editing of the manuscript; critical literature review; intellectual participation in the propaedeutic and/or therapeutic conduct in the studied case.

Ricardo Romiti: Study conception and planning; drafting and editing of the manuscript; critical review of the literature; intellectual participation in the propaedeutic and/or therapeutic conduct in the studied case.

## Conflicts of interest

None declared.
